# A Modeling Study on How Cell Division Affects Properties of Epithelial Tissues Under Isotropic Growth

**DOI:** 10.1371/journal.pone.0011750

**Published:** 2010-07-30

**Authors:** Patrik Sahlin, Henrik Jönsson

**Affiliations:** Computational Biology and Biological Physics, Department of Astronomy and Theoretical Physics, Lund University, Lund, Sweden; University of Nottingham, United Kingdom

## Abstract

Cell proliferation affects both cellular geometry and topology in a growing tissue, and hence rules for cell division are key to understanding multicellular development. Epithelial cell layers have for long times been used to investigate how cell proliferation leads to tissue-scale properties, including organism-independent distributions of cell areas and number of neighbors. We use a cell-based two-dimensional tissue growth model including mechanics to investigate how different cell division rules result in different statistical properties of the cells at the tissue level. We focus on isotropic growth and division rules suggested for plant cells, and compare the models with data from the *Arabidopsis* shoot. We find that several division rules can lead to the correct distribution of number of neighbors, as seen in recent studies. In addition we find that when also geometrical properties are taken into account other constraints on the cell division rules result. We find that division rules acting in favor of equally sized and symmetrically shaped daughter cells can best describe the statistical tissue properties.

## Introduction

Multicellular development is governed by cellular differentiation and morphogenesis. Cellular differentiation has mainly been described as a process of gene regulation and molecular signaling between cells, although signaling via mechanical interactions due to the morphogenesis has recently been suggested [1]–[4]. Both molecular and mechanical signaling between cells in a growing tissue are affected by cell division. Therefore, cell division is one of the means for an organism to regulate different aspects of development [5].

In many growing epithelial tissues, cells divide perpendicular to the surface and this allows for a detailed study of cell topology (quantified by the number of neighbors for each cell) and geometry (cell shapes and sizes) in these monolayered tissues. Such a tissue may hence be described as a two-dimensional sheet defined by vertex points representing wall junctions, one-dimensional edges representing cell walls, and two-dimensional faces representing cells. Epithelial tissues are dominated by three-cell vertices and according to Euler's law the average number of neighbors is therefore equal to six. In the 1920's, F.T. Lewis showed that cucumber epithelium has a skew distribution of number of neighbors, dominated by hexagonal cells (47%) and with more five-sided cells (25%) than seven-sided (22%) [6], [7]. He also noted that the distribution was quite narrow, ranging from four- to eight-sided cells. More interestingly, surprisingly similar topologies have been found in epithelia of many species ranging over different kingdoms [8]. An important question is how these topological distributions can emerge at a tissue level from cell division.

The epidermal layer in plants provides a beneficial model system for investigating cell division without cellular reorganization, since plant cell walls govern tissue rigidity and there is no sliding between cells. Hence, cell division is the only way to affect the topology of the tissue and proper cell division is needed for developmental processes in the plant [5]. When a plant cell divides, a new cell wall is added between the two daughter nuclei. In the epidermal cell layer new walls are anticlinal, preserving the two-dimensional structure of the tissue. Also, at the shoot apical meristem (SAM) summit, growth is isotropic [9], [10], and the tissue may be represented by a two-dimensional sheet with isotropic growth.

Rules for determining the position and direction of new cell walls in plants have been proposed for more than a century [5], [11]–[14]. Hofmeister (1863) suggested that cells divide perpendicular to the main axis of growth, which also correlates with the main axis of cell extension in many plant tissues. Sachs (1878) noted that new walls form nearly perpendicularly to older walls. Errera (1888) proposed that cells behave similarly to soap bubbles, and that cells are divided by the shortest path dividing the cells into two equally sized daughters. More recently, cell growth and proliferation have been investigated in more detail at the plant shoot, and while clear directional patterns can be found at the periphery where new organs form, strain is isotropic and proliferation directions are omnidirectional at the apex [9], [10]. Division planes in mother and daughter cells can be related where orthogonal division directions are common [9], [10]. Recently, a correlation between the directions of cortical microtubules (MTs) and the cell division plane has also been found [4], [15]. At the SAM summit the MT directions are dynamic and suggested to be random [4]. Two main rules for orienting MTs in plants have been proposed; perpendicular to maximal strain directions, and parallel to maximal stress directions [4], [16].

What biological mechanisms determine positions and directions of cell division are still unknown, and it may very well be that different mechanisms act in different organisms and even in different tissues of the same organism. Cell division rules have been investigated in mathematical models for a long time [14]. Mathematical models of cell division have recently been used to show that different division rules lead to specific topological distributions on a tissue-scale, and that a subset of the division rules successfully reproduce the common topology distribution found in the epithelium of several organisms [8], [17]. These models have neglected geometrical properties, but an important property of the successful models was symmetric cell division, *i.e.* the vertices of the mother cell are distributed evenly among the daughter cells.

We have previously introduced a two-dimensional spring-based model to take also geometrical aspects into account and compared simulated tissues with data from the *Arabidopsis* SAM [18]. We were able to show that although cell wall-mechanics is not important for the resulting topology, simulations with mechanics resulted in better shaped cells. Here we continue to use the spring-based model to test different division rules and compare results with experimental data. Using the spring-based model we are able to investigate consequences for both topology and geometry of using the different division rules in an isotropically growing epidermal tissue.

### Definitions of division rules and tissue properties

At cell division the mother cell is divided into two daughter cells by introducing a new wall, which is described by a *division plane*. How the division plane is located is determined in the model by a *division rule*. A division rule consists of two mechanisms; one to determine the *division center* and one to determine the *division direction*. The division plane is then the straight path that goes through the division center parallel to the division direction.

In this work we are studying two different mechanisms for determining division centers,

- Center of Mass (COM). The division center is the center of mass of the mother cell.
- Random. The division center is a random point within the mother cell drawn from a uniform distribution.

The COM rule will produce daughters with quite symmetric sizes, while the Random rule allows for asymmetrically sized daughters. In addition, we are studying four different mechanisms for determining the division direction,

- ShortestPath. The direction is the shortest path through the division center. Combined with the COM mechanism for determining the division center this is our representation of Errera's rule [13].
- RandomDirection. The direction is randomly chosen from a uniform distribution.
- Orthogonal. The division direction is orthogonal to the direction of the previous cell division, following patterns seen in plant tissues [9], [10].
- StrainPerpendicular. The direction is perpendicular to the direction of strain in the mother cell (Methods). This rule is our representation of Hofmeister's cell division rule [11].

The division directions are important for determining the shapes of the daughter cells, where the ShortestPath favors more symmetrically shaped daughter cells, while RandomDirection has no such bias. The two types of mechanisms are combined into division rules with the following notation DivisionDirection 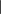
DivisionCenter.

We are interested in two different types of tissue properties.

- **Topology**. We quantify the topology of the tissue by the distribution of number of neighbors.
- **Geometry**. We quantify the geometry of the tissue by distributions of cell shapes and sizes.

## Results and Discussion

We performed series of simulations with isotropic growth using a two-dimensional spring-based model, and with different division rules (Methods, Introduction). Cells outside a boundary were removed and statistics was gathered from snapshots of simulated tissues at different time points, neglecting cells at the boundary of the tissue. We analyzed the topologies and the geometries of simulated tissues and compared with experimental data to test different division rules. We also investigated how well tissues fitted to Lewis' law, which states that a linear relationship exists between number of neighbors and areas of cells [7]. Finally, we also simulated an oryzalin experiment by continuing tissue growth after suspending cell division [19].

### The COM mechanism for determining the division center is superior to the Random mechanism in reproducing the topology of experiments

**Figure 1:**
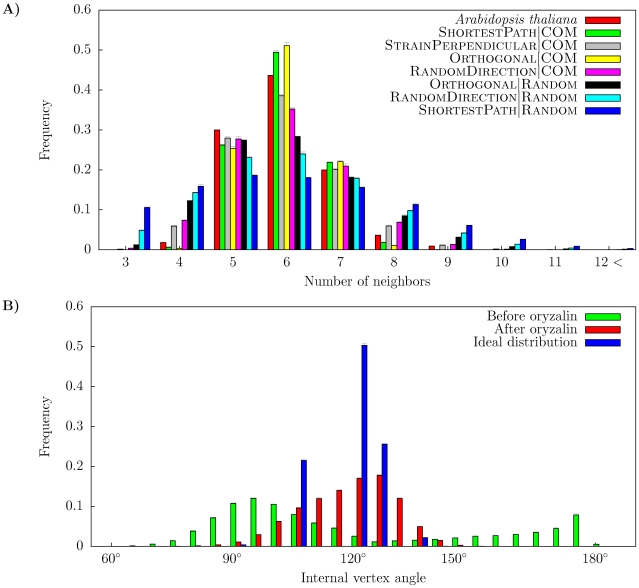
Distributions of number of neighbors and internal vertex angles from simulations with different division rules. Error bars represent standard deviation. A) Distributions of number of neighbors. Experimental data from *Arabidopsis thaliana* is also presented for comparison. B) Distributions of internal vertex angles before and after suppression of cell division. For comparison, the ideal distribution of internal vertex angles if all cells were regular polygons is plotted.

**Figure 2:**
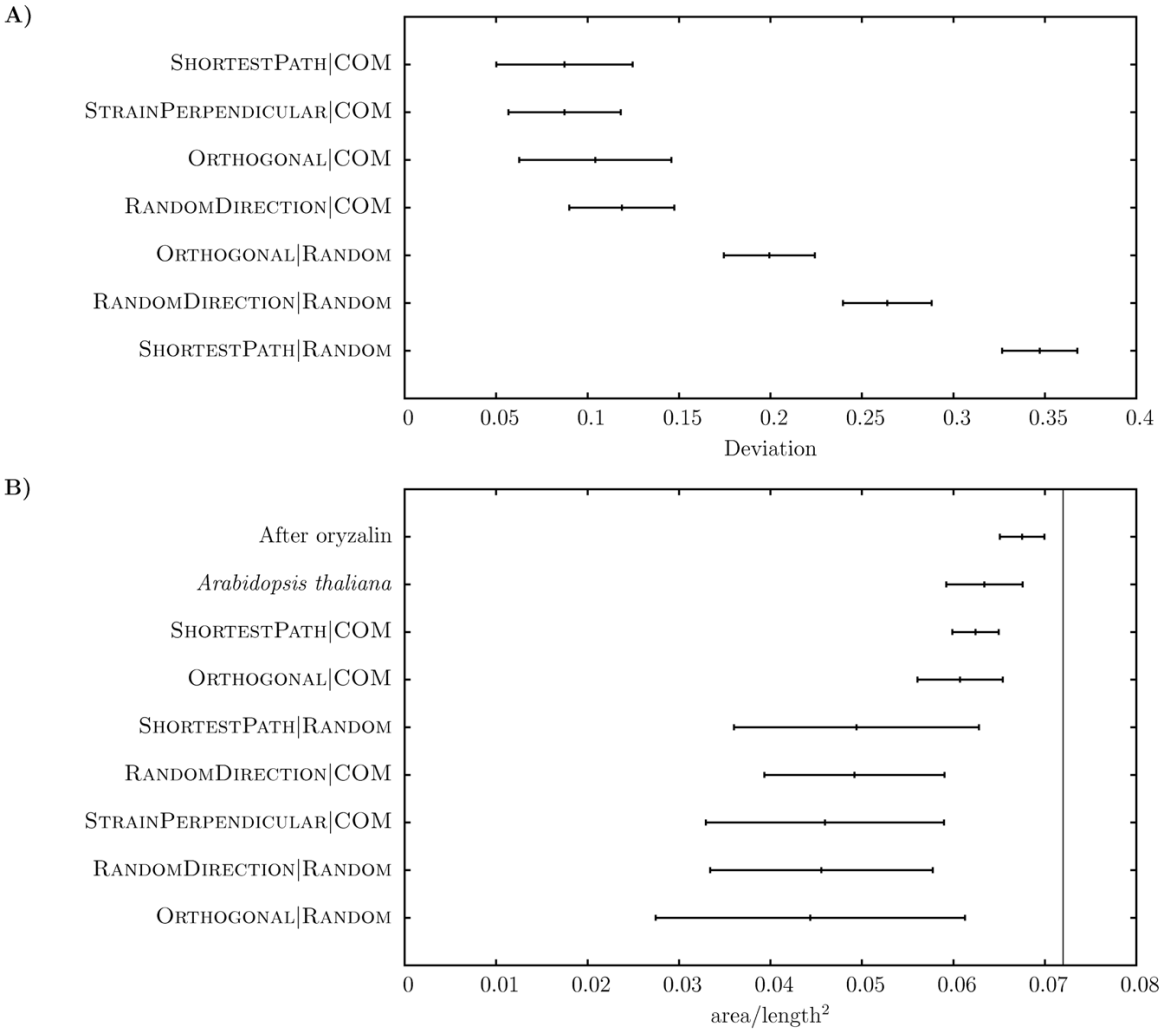
Topological and geometrical properties of simulated tissues. A) The deviation of each division rule. The deviation quantitatively measures how well resulting tissues of simulations with a given division rule reproduces the distribution of number of neighbors compared with experimental data of *Arabidopsis thaliana*. B) Results from the quantitatively measurement of cell shape (Methods). The numerical values for the shape measurement range from zero (“flat” cells without area) and 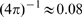 (circular cells). The vertical line marks the value 0.072, which is the approximate value corresponding to a regular hexagon.

First we studied topologies resulting from simulations with the different division rules and compared them with data from the *Arabidopsis* SAM (Figure 1A, Table 1). It can be seen that all division rules using the Random mechanism for the orientation of the division center generate distributions of number of neighbors that were wider than the one for the experimental data. To quantify the difference between the topology resulting from each model and the experimental topology, we defined a deviation measure (Methods, Fig. 2A). As can be seen, there is a clear separation between models using the COM mechanism and models using the Random mechanism, where the former have a lower deviation. One interesting feature of the experimental number of neighbor distributions is the skewness. Although all models display a skewness in the distribution of number of neighbors, where the number of five-neighbor cells is always larger than the number of seven-neighbor cells (Fig. 1A), the skewnesses associated with models using the COM mechanism are weaker compared with experimental data (Table 1).

**Table 1:** Standard deviation and skewness of distributions of number of neighbors.

	Number of neighbor distributions.	
Division rule	Standard deviation	Skewness
Orthogonal 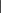 COM	0.73 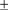 0.04	0.14 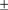 0.13
ShortestPath 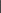 COM	0.76 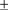 0.04	0.20 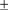 0.14
*Arabidopsis thaliana*	0.90	0.53
StrainPerpendicular 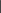 COM	1.0 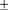 0.0	0.39 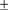 0.20
RandomDirection 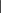 COM	1.1 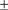 0.1	0.29 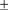 0.17
Orthogonal 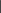 Random	1.4 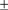 0.1	0.54 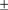 0.16
Random 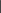 Random	1.6 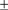 0.1	0.41 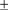 0.13
ShortestPath 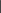 Random	1.9 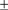 0.1	0.41 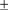 0.08

Standard deviation and skewness have been measured for each simulated tissue and the values presented are average values with standard deviation as errors.

Among the rules using the COM mechanism, there was a slight advantage for the ShortestPath 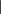
COM and the StrainPerpendicular 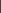
COM division rules. An interesting result is that the deviation is not fully correlated with the mechanism for determining the division direction. While the ShortestPath 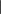
COM division rule had lowest deviation, the ShortestPath 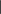
Random division rule generally performed badly. This shows how important a proper mechanism for determining the division center is as a top performing mechanism for orienting the division direction can easily be turned into the worst by changing the mechanism for determining the division center.

In conclusion, our results show that division rules that divide mother cells into almost symmetrically sized daughters result in topologies with lower deviation from experimental data than division rules that generated more asymmetric daughter sizes. This feature was particularly important for generating narrow distributions – as seen in the experimental data – and agrees with what has been shown with non-geometrical models for which a symmetric division of cell vertices has been shown to be important for narrow distributions [8], [17].

### ShortestPath and Orthogonal division directions produce most plant-like cell shapes

**Figure 3:**
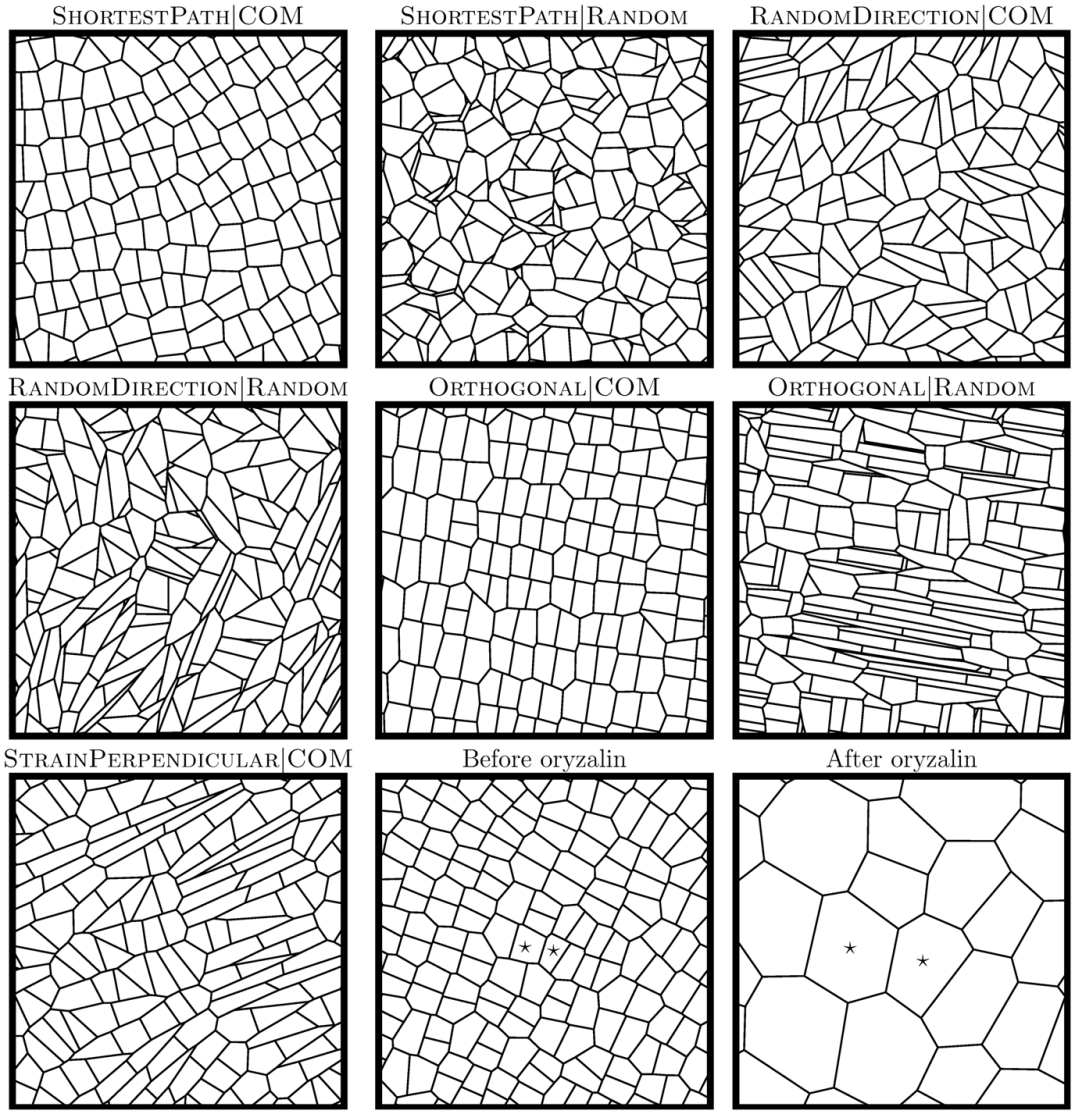
Example images of tissues from simulations with different division rules. The stars in the tissues from the oryzalin experiment identify cells before and after cell division has been suspended. The width and height of all images are ten length units.

For simulated tissues generated by division rules with symmetric division (using the COM mechanism), topology alone could not be used to discern among the division rules studied in this work. Instead, we also analyzed geometrical properties, where one can note that cells at the *Arabidopsis* apex have quite symmetrical shapes as measured by the cell area divided by the total cell wall length squared [18]. This shape measure revealed that – for all proposed division rules – cells of the SAM were more symmetrically shaped than cells of simulated tissues (Fig. 2B). Cell shapes of each division rule were differently shaped (Fig. 3 for examples). The division rules that generated cell shapes closest to the experimental data were those with systematic rules for dividing the cell such that the wall is directed perpendicularly to the main axis of the cell (Fig. 2B). The ShortestPath division rule does this explicitly, and the Orthogonal division rule does it implicitly since the isotropic growth together with cell division perpendicular to the last division plane will approximate the shortest path. Interestingly, both these rules have been suggested for plant cells. A third division rule suggested for plant cells is to divide the cell perpendicular to the principal strain direction, but with isotropic growth the maximal growth direction is ambiguous, and the rule led to cell shapes similar to those obtained by choosing a random division direction (Fig. 2B).

In conclusion, our results suggest that epithelial cells in an isotropically growing tissue tend to divide such that symmetrically shaped daughter cells are favored. Our results also emphasize that while using a given division rule can result in a topology very similar to what is found in experiments, the same division rule might not correctly reproduce geometrical properties, in this case cell shape. For example, using the StrainPerpendicular 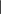
COM division rule results in a topology resembling the one of an *Arabidopsis* meristem, but the same series of simulations produce cells with a different shape distribution compared with the experimental data. A final note is that an absolute requirement for any of the division rules to generate cell shapes similar to what is seen in the experimental SAM data is that the walls have mechanical properties [18].

### Analysis of cell size distributions via Lewis' law reveals small discrepancies between the models and the data

**Figure 4:**
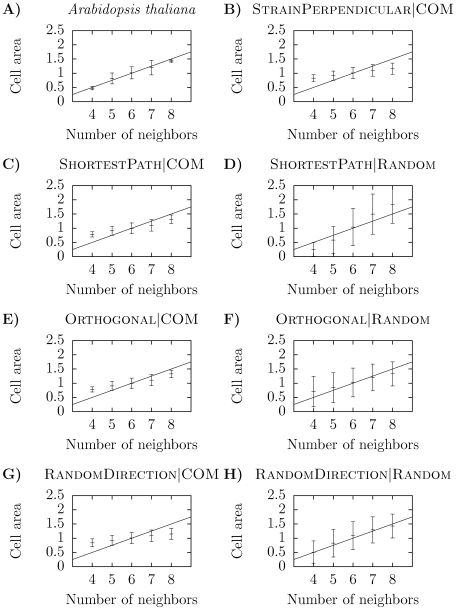
Cell area plotted as a function of number of neighbors for different division rules. Cell areas are normalized such that the average cell area – including all cells of the tissue – is equal to unity. Presented data is average values together with standard deviations. The diagonal line is the relationship: 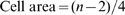, where 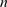 is number of neighbors, defining Lewis' law [7].

Another geometrical property of the tissue is cell sizes. We used Lewis' law [7] – stating that a linear relationship exists between number of neighbors and areas of cells – to compare the distributions of cell areas from our simulations with the distribution of the SAM (Fig. 4). The experimental data displayed an almost perfect fit to the linear function defined by Lewis' law. The data from the simulations showed a linear dependence between number of neighbors and areas, but the slope deviated slightly from Lewis' law. For example, the two best performing models sofar – ShortestPath 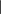
COM and Orthogonal 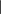
COM – have a lower slope compared with Lewis' law. This is an indication that the COM positioning mechanism may generate too symmetrically sized daughters, and will hence not allow for as large deviations in cell area as seen in experiments. Partly, this result depends on our definition of a constant maximal cell size in the model (Methods). If the daughter cells are equally sized, the resulting tissues will have cell areas bounded below by a factor one half of this maximal area. But the ShortestPath 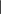
COM and Orthogonal 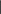
COM models also have narrower distributions of number of neighbors compared with experiments (Table 1), which is another indication that the cell divisions of the models are too symmetric. In the plant cell the position of the division plane is guided by the nucleus, which is often located centrally in the cell, although not exactly at the center of mass. Hence, the positioning of the division plane may be at a random position close to the center of mass, which can be interpreted as something in-between our COM and Random positioning mechanisms. Interestingly, this may increase the slope of the cell areas as a function of number of neighbors for the ShortestPath division rule (cf. Figs. 4C and D) while this may not be the case for the Orthogonal division rule (cf. Figs. 4E and F).

### The tissue model qualitatively reproduces the behavior of an experiment where cell division is impaired

The microtubules of the shoot meristem can be depolymerized by application of oryzalin [19]. In the experiments lack of microtubules resulted in inhibited cell division. Cells still grow and the internal vertex angles converge towards 120.

We performed a series of simulations without cell division to test our model for this perturbation experiment. First we performed a first series of simulations with the ShortestPath division rule and then, using the tissues from the first series as initial states, we performed a second series of simulations, but this time without cell division. The angular distributions in the non-dividing case clearly changed and peaked close to 120 (Fig. 1B) Example images of tissues from simulations before and after suspension of cell division are presented in Fig. 3.

Our model does not allow for curved walls. Therefore we did not expect all internal vertex angles to converge towards 120. The sum of all internal vertex angles of a cell is equal to 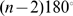, where 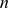 is the number of neighbors, so if the cell takes the shape of a regular polygon the internal vertex angle of each vertex is equal to 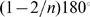. We calculated the ideal distribution of internal vertex angles – *i.e.* when all cells are regular polygons – to compare with the distribution resulting from simulations without cell division. The distribution of internal vertex angles for simulated tissues converged towards this ideal distribution after cell division was disabled (Fig. 1B).

We also compared the shapes of cells before and after disabling cell division in the model (Fig. 2B). Cells in simulated tissue before division is suspended are less symmetrically shaped than cells in the unperturbed experiment, but cells after division is suspended are more symmetrically shaped than cells in the experiment, showing that cell divisions act “against” symmetrically shaped cells.

### Conclusions

Already in the 1920s F.T. Lewis noted statistical properties of the topology and geometry of epithelial plant tissue, which later have been seen also in other species. At the same time, the discussion on rules for determining cell division planes in plants has been ongoing since the 19th century. We have used a model of a two-dimensional growing tissue which includes mechanical properties to test several of these rules against experimental data of topological and statistical properties of the epidermal layer of the shoot apical meristem of *Arabidopsis thaliana*.

Our results suggest that epithelial cells in an isotropically growing tissue tend to divide such that the daughter cells are symmetric both in size and in shape, which depends on positioning a new wall close to the center of the cell and to divide along some approximation of a shortest path. It is well known that a shortest path rule for dividing plant cells is not a general rule of generating division directions. Examples exist for which this rule performs badly, *e.g.* in the boundary region between the shoot apical meristem and forming primordium [4]. It was suggested that the microtubules in these regions align in directions following the principal stress direction, and the divisions tended to be along this direction, independent of cell shape. It may very well be that different mechanisms interact and that in regions of isotropic growth, where stresses are isotropic, a shortest path rule might be the result.

Although we have focused our investigations on isotropically growing tissues, a simple and interesting extension would be to also investigate the division rules in anisotropically growing tissues where there might be a clearer separation of the results for different division rules, given that there are less symmetric cells.

We have shown that statistical comparisons are useful when comparing different division rules, and pointed out some features that are important to achieve correlation with the experiments. However, a statistical approach will not always be able to discern among competing hypotheses since several can lead to the same statistical distributions. By comparing the models with live imaging data it will be possible to test different division rules at a cellular level.

Models – such as the one presented here – will be essential to be able to compare rules not only depending on the cell itself, but also for testing hypotheses based on variables depending on the tissue neighborhood such as growth and mechanical-based mechanisms.

## Materials and Methods

### The model

The two-dimensional spring-based model is a mechanical model for the epidermal layer of plant tissue. Cells are represented by vertices connected by edges representing cell walls. The edges are treated as mechanical springs and give the mechanical properties of cell walls. The vertices are treated as being in a viscous medium; their velocities are proportional to the forces acting upon them.

The contribution of forces from walls acting on vertex 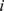 is
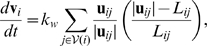
(1)where 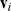 is the position of vertex 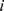, 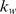 is a constant that determines the stiffness of walls, 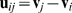, and 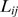 is the resting length of the wall connecting vertices 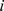 and 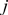. The summation is over all vertices connected via edges to vertex 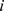.

Cell walls grow under tension. Resting lengths of cell walls are in the model increased as walls are being stretched,
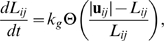
(2)where 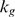 is a constant that sets the rate of growth and 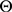 is the ramp function defined as
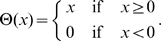
(3)


For the StrainPerpendicular division rule the direction of division is parallel to the strain pattern of a cell. We calculate the direction of strain of a cell using circular statistics according to
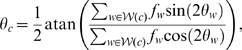
(4)where 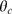 is the direction of strain of cell 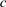, 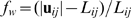 is the magnitude of strain of wall 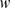, and 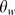 is the direction of wall 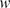. The summation is over all walls of cell 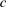.

The focus of this work is to model the development of the shoot apical meristem. Turgor pressure and internal growth is represented by a radial force,
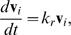
(5)where 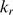 is a constant which determines the internal growth rate. The model is an approximation of the meristem and the further cells are located from the origin the less accurate is the representation of cells in the epidermal layer. Cells outside a threshold radius, 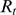, are therefore removed.

### Cell division

A cell is divided into two daughter cells if its area exceeds a threshold value, 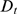. The division plane is defined by a spatial position and a direction. The division plane is then the straight path that passes the spatial position in the given direction. A division rule determines how the division plane is located (Introduction). At each cell division two new vertices are added to two walls of the mother cells and a new cell wall is added connecting these two vertices. The resting length of the new cell wall is set to be equal to the distance between the two vertices. The two original walls of the mother cell are each split into two new walls. The resting lengths of the new walls are set proportionally to respective length such that 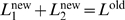, and 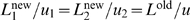, where 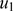 and 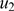 are the lengths of the two new wall segments and 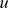 is the length of the old wall. If the distance between a new vertex and a three-vertex is shorter than a threshold, 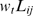, then the vertex is moved to the threshold position. This measure is taken to avoid four-vertices.

### Numerical simulations

A fifth-order Runge-Kutta ODE solver using adaptive stepsize is used for all simulations. The initial states that are used in the simulations are obtained in the following way. First an initial state of one single cell is created. This single cell is then used in a longer simulation and from this simulation 25 snapshots of the tissue are captured and stored to be used as initial states. This process is repeated with 13 different initial single cells represented by regular polygons of 3 to 15 vertices. In total 325 initial states are created and used in the simulations of each division rule. The ShortestPath 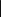
COM division rule is used in the simulations to generate initial states as the division rule has in previous studies proved itself to generate plant-like tissues [18].

The average numbers of cells (with standard deviations) at one snapshot in simulated tissues were; 237
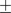
45 (ShortestPath 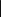
COM), 437
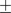
66 (ShortestPath 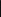
Random), 231
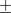
43 (RandomDirection 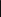
COM), 310
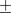
48 (RandomDirection 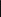
Random), 235
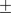
42 (Orthogonal 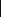
COM), and 282
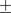
46 (Orthogonal 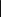
Random), 218
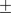
39 (StrainPerpendicular 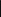
COM). Cells on the boundary of the tissue were neglected.

Parameter values are presented in Table 2. We have performed a robustness analysis by performing series of simulations with parameter values perturbed by an order of magnitude. The analysis showed that the results are robust to these parameter perturbations (data not shown).

**Table 2:** The different sets of parameter values that was used in the different types of simulations.

	Model parameters.	
Parameter	Standard simulations	Oryzalin simulations
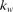	0.05	0.05
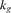	0.01	0.01
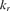	0.05	0.05
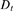	1.0	-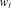	0.1	-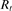	7.0	-

### Experimental data

We compare simulations of the model with experimental data from *Arabidopsis thaliana*. The experimental data consists of a tissue with 110 cells and is taken from [18].

### Data analysis

After each simulation data is gathered from the resulting tissue. There are three types of data that are gathered; number of neighbors, internal vertex angles, and a measurement of cell shape. While gathering data we neglect cells at the boundary since such cells can be affected by the boundary condition.

The measurement of cell shape is here defined as the ratio between cell area and the total length of cell walls squared. The numerical value of this measurement ranges from zero for cells without area, to 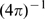 for circular cells.

We introduced a deviation measure to quantify how well a simulated tissue reproduces the distributions of number of neighbors from experiments. The deviation measure is defined as
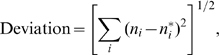
(6)where 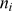 is the fraction of cells in the tissue with 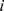 neighbors, and 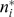 is the corresponding fraction measured from experimental data.
